# Cuba’s Two Meals a Day

**Published:** 1888-12

**Authors:** 


### CUBA'S TWO MEALS A DAY.

Only two meals a day are served in Cuban hotels. They live much as people do in some parts of France and Switzerland. You take an orange or two with a cup of coffee and a roll in the early morning ; a liberal breakfast, in courses is served at 11 o'clock, and a ceremonious dinner at about 4 or 5 in the afternoon. This mode of living is admirably suited to the climate, and you fall in with the custom and like it at once. The breakfast opens with small olives and fresh radishes served in the same dish ; the next course is fish, then eggs meat, etc. You are not asked what you prefer, but each course is set before you and you can partake of it or not.

Instead of beginning with fruit, the Cuban breakfast ends with it—pineapples just cut from the stock, bananas freshly picked, sapodillas, a faint and rather over-sweet morsel, with oranges *ad libitum*. In Florida and many other parts of the country the orange is cut in halves, and its juice and pulp are passed to the mouth with a teaspoon. In Havana the orange is served whole on the table, peeled down to the juicy "meat of the fruit," and you present the golden ball to your lips on the prongs of a fork.

At any and every American hotel the moment you sit down the question is almost flung at you, "tea or coffee ?" Cubans better understand what is healthful. They follow nature's plan and take their meals more as the lower animals do. Cubans do not fill up their stomachs with fluids during meals.—*Ex.*

